# Macrophage Reprogramming and Cancer Therapeutics: Role of iNOS-Derived NO

**DOI:** 10.3390/cells10113194

**Published:** 2021-11-16

**Authors:** Khosrow Kashfi, Jasmine Kannikal, Niharika Nath

**Affiliations:** 1 Department of Molecular, Cellular, and Biomedical Sciences, Sophie Davis School of Biomedical Education, City University of New York School of Medicine, New York, NY 10031, USA; kashfi@med.cuny.edu; 2 Graduate Program in Biology, City University of New York Graduate Center, New York, NY 10016, USA; 3 Department of Biological and Chemical Sciences, College of Arts and Sciences, New York Institute of Technology, New York, NY 10023, USA; jkannika@nyit.edu

**Keywords:** nitric oxide, iNOS, tumor-associated macrophage, M1, M2, miRNA, arginase, cancer progression

## Abstract

Nitric oxide and its production by iNOS is an established mechanism critical to tumor promotion or suppression. Macrophages have important roles in immunity, development, and progression of cancer and have a controversial role in pro- and antitumoral effects. The tumor microenvironment consists of tumor-associated macrophages (TAM), among other cell types that influence the fate of the growing tumor. Depending on the microenvironment and various cues, macrophages polarize into a continuum represented by the M1-like pro-inflammatory phenotype or the anti-inflammatory M2-like phenotype; these two are predominant, while there are subsets and intermediates. Manipulating their plasticity through programming or reprogramming of M2-like to M1-like phenotypes presents the opportunity to maximize tumoricidal defenses. The dual role of iNOS-derived NO also influences TAM activity by repolarization to tumoricidal M1-type phenotype. Regulatory pathways and immunomodulation achieve this through miRNA that may inhibit the immunosuppressive tumor microenvironment. This review summarizes the classical physiology of macrophages and polarization, iNOS activities, and evidence towards TAM reprogramming with current information in glioblastoma and melanoma models, and the immunomodulatory and therapeutic options using iNOS or NO-dependent strategies.

## 1. Nitric Oxide and iNOS

The tumor and the immune cell interaction are critical areas of study in cancer growth and progression. Macrophages have a controversial role in the tumor microenvironment with both anti- and pro- tumoral effects [1]. The gaseous transmitter and signaling molecule nitric oxide (NO) and the expression of inducible nitric oxide synthase (iNOS) that produces NO are noted to have dual roles in cancer, which is to either promote or inhibit tumor growth [2,3]. Therefore, NO shows potential either as a therapeutic agent in its own right or as a target molecule in cancer therapies.

NO is a small signaling molecule that is synthesized by three NO synthases (NOS) isoforms. The two isoforms neuronal NOS (nNOS or NOS I), and endothelial NOS (eNOS or NOS III) are constitutive. The third NOS isoform (NOS 2 or inducible NOS, iNOS) is negligible in resting cells and is induced by cytokines and bacterial lipopolysaccharide (LPS) [3,4]. NO plays an important role in cell growth, differentiation, and apoptosis [3,5]. The enzyme iNOS produces NO through the conversion of L-arginine into citrulline utilizing NADPH and oxygen. NO may also be associated with resistance to apoptosis [6] and immune escape [7]. iNOS is induced by inflammatory cytokines [8] and is transcriptionally regulated [9]. It is well established and acknowledged that NO’s role in cancer depends on its concentration, exposure duration in cells, cell-specific sensitivities, iNOS localization in tissues, and extracellular conditions [10,11,12].

Antitumor effects of NO have been demonstrated even though its pro-tumor effects have largely dominated the scenarios. In terms of the tumorigenic effects, NO contributes to tumor growth and metastasis, regulates metabolism by the Warburg effect, and promotes cancer growth via high glycolytic activity [13]. Furthermore, antitumor iNOS activity is related to its cytotoxicity and immunogenic effects [14]. Therefore, NO-releasing hybrids are subjects of intensive investigations as potential anticancer drugs, either as single cytotoxic agents or in combination with standard radio- and chemotherapy [13].

More recently, studies relate the chemo- and immunoresistance [7] in cancer cells with NO as a mediator for the events in the tumor microenvironment (TME) and as a bonafide molecular target [15,16]. This review summarizes the classical physiology of macrophages and polarization, iNOS activities, and presents evidence towards tumor-associated macrophage reprogramming and the various immunometabolic and therapeutic options using iNOS or NO-dependent strategies.

## 2. Macrophage and Plasticity

As the principal biological modulators of the mammalian immune response, macrophages represent a heterogeneous population of predominantly mononuclear leukocytes that demonstrate significant variability in location, morphology, and function [17]. Historical conventions have generally associated macrophage nomenclature with its host organ; for instance, these cells have been distinguished as microglia in the brain, Kupffer cells in the liver, intraglomerular mesangial cells in the kidney, and Langerhan cells in the skin, among other varieties [18]. Collectively regarded as both the local and systemic effectors of the inflammatory response, these white blood cells are often distinguished according to their motile and stationary properties, which give insight into their developmental stage, their function at any specific point in time, and an organism’s overall homeostatic state [19].

Prior to discussing the functional divergence of macrophages, it is vital to first address its molecular divergence by distinguishing between embryonic-derived and monocyte-derived macrophage subtypes. Traditional hypotheses have proposed that tissue-resident macrophages evolved primarily from freely-circulating monocytes [20,21] and evidences also point to embryonic precursor cells as the principal origin of mature macrophages [22]. More recently, it is understood that each tissue has its own composition of embryonic-derived and adult-derived macrophages, with studies continuing to examine if such macrophages of distinct origins are unique in their roles or functionally interchangeable at any point [23]. The theoretical foundation of the monocyte derived developmental pathways centers on hematopoietic stem cells in the bone marrow, giving rise to naive monocytes, which are either channeled to the bloodstream or stored in the spleen for future generations’ mobilization. In the event of infection or injury, these monocytes are recruited to the specific site of stress, where they mature and mediate higher-level immune functions, including phagocytosis and inflammatory cytokine cascades [24]. Broadly termed “histiocytes”, these docked macrophages, by definition, denote mature myelophagocytic cells that have migrated to various tissular sites throughout the body, aggregating to form more specialized immune cell subpopulations.

In contrast, the embryonic precursor morphological course omits the necessity of a pathological trigger to incite further maturation. In this scheme, the extraembryonic yolk sac gives rise to tissue-resident macrophages directly or via fetal liver monocyte intermediates [21]. Based on variations in transcription factors and cell-surface markers, macrophage ontogeny can be profiled with relative accuracy. The appropriate molecular characterization, in turn, helps researchers conclude whether the population being studied possesses self-renewing properties, as in the case of embryo-derived macrophages, or whether they are terminally differentiated, following the monocyte-derived developmental fate [22].

Despite their variability in terms of lineage, macrophages are most commonly classified by their physiological function. Although macrophages have various roles, they are typically designated classically-activated M1 or alternatively-activated M2 macrophages [18,25]. M1 macrophages exist as pro-inflammatory cells responsible for initiating an immune response, compromising tissue integrity, and mitigating tumorigenicity overall. This cellular behavior diverges from their M2 analogs, which exist as anti-inflammatory cells that participate in immunosuppressive processes, tissue remodeling, and promoting tumor development in general [20,26]. Although their phenotypical fate depends on signals from the microenvironment, these effector cells exhibit high plasticity and, therefore, may transition between antagonistic conformation in response to peripheral cues. As key modulators of local and systemic inflammatory responses, this macrophage duality has recently emerged as an attractive target for therapeutic interventions against cancer.

### 2.1. M1-like Phenotype

M1 macrophages are responsible for ongoing immune surveillance against pathogenicity. Upon activation by the presence of microbial lipopolysaccharide (LPS) [27] or LPS in conjunction with T-helper 1 (Th1) cytokines, the most notable of which is interferon- γ (IFN-γ), there occurs the release of pro-inflammatory interleukins. These are IL-1β, IL-6, IL-12, IL-23, TNF-α, and additionally, chemokines, hydrolyzed proteases, interferons, and reactive oxygen species (ROS) that are released downstream as part of the innate immunity response [28,29]. As part of this microbicidal activity, M1 macrophages trigger NOS and NO production. To amplify these effects, M1 macrophages limit nutrients, such as iron, and promote phagosome acidification to defend against harmful microorganisms [30]. In addition, classically-activated M1 macrophages actively participate in rampant antigen-processing and subsequent presentation through gene upregulation. This elicits a more robust T-cell response while engaging the adaptive immune system as part of a more comprehensive defense.

The second phase of M1′s multipronged immune response involves the promotion of localized tissue damage. Classically activated macrophages synthesize NO as a byproduct, inhibiting cell proliferation and inducing oxidative stress at high concentrations. While this poses a potential threat to healthy tissues, the cytotoxic conditions that result equally have the potential to kill tumor cells, effectively inducing apoptosis via either the LAK cells, tumor-targeting lymphocytes, or NK cells. In this context, NO possesses the ability to directly damage DNA while inhibiting DNA synthesis and ribonucleotide reductase activity [31]. More indirect mechanisms include the attenuation of cis-aconitase enzymatic function, partial depletion of vital iron stores, reduced oxygen consumption, and interference with complexes I and II of the electron transport chain [32,33]. Although TNF-α also induces similar effects, it is acknowledged as a secondary deterrent of angiogenesis and metastasis.

### 2.2. M2-like Phenotype

**Figure 1:**
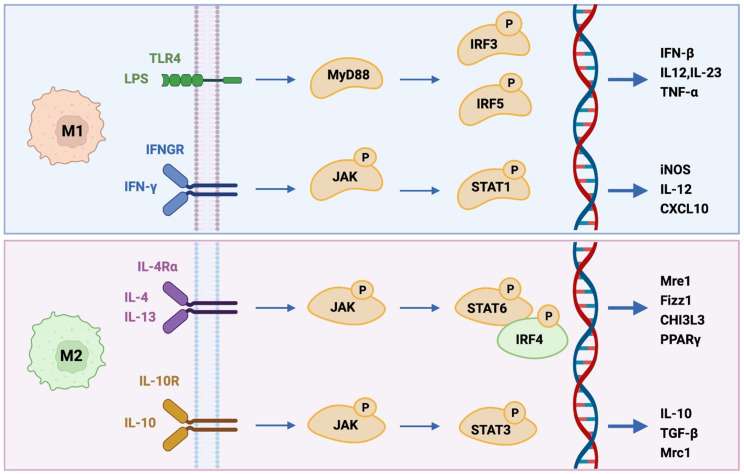
Major M1 and M2 differentiation pathways. Briefly, M1-like macrophages (upper panel) can be induced by IFN-γ and LPS-mediated activation of TLR-4 signaling pathway, promoting inflammatory responses by secreting cytokines such as TNF-α, IL-1α, IL-1β, IL-6, IL-12, IL-18, and IL-23. Alternatively, IL-4 and IL-13 induce macrophages to M2-like (lower panel) by activating STAT6 via the IL-4 receptor alpha (IL-4Rα), whereas IL-10 promotes M2 phenotype by activating STAT3 through IL-10R. In the IL-4 and IL-13 pathway, receptor binding of IL-4 activates JAK1 and JAK3 leading to STAT6 activation and translocation.

In contrast, alternatively-activated M2 macrophages assume a predominantly anti-inflammatory role, attenuating the host immune response [34]. Briefly, IL-4 and IL-13 polarize macrophages to M2 phenotype by activating STAT6 by the IL-4 receptor alpha (IL-4Rα), while IL-10 promotes M2 phenotype via STAT3 through receptor (IL-10R), (Figure 1). The M2 class of macrophages have been segmented into M2a induced by the T-helper 2 (Th2) cytokines IL-4 or IL-13, M2b produced by immune complexes, LPS, IL-1β and M2c that are produced by glucocorticoids, IL-10 or TGF-β [35]. Additionally, murine M1 macrophages show IL-4R-independent phenotype switch to M2d-like, which produce VEGFand IL-10 [36]. True to its M1 paradoxical nature, evidence has shown that IL-4, which gives rise to M2a, behaves inversely to IFN-γ as it works to downregulate NOS expression, thus impairing NO synthesis [35]. Macrophage activation nomenclature updates in the last decade identify the activation signal leading to differentiation. According to this modern nomenclature M2-like macrophage subsets induced by IL-4, immune complexes or IL-10 are named M(IL4), M(Ic), and M(IL-10) respectively, and exhibit anti-inflammatory properties that are equated to the M2a, M2b, and M2c like phenotypes [37].

Similarly, a key biomarker of M2 activation is elevated levels of arginase-1 (Arg-1) which breaks down arginine and further adversely impacts the production of NO, as less substrate becomes available. Arg-1 converts L-arginine to urea and ornithine, which is used in the urea cycle [24,38]. In addition, the metabolites, urea, and ornithine sustain regenerative processes such as wound healing, cell replication, and angiogenesis as opposed to NO or ROS produced in the M1 pathway. Compounding this, M2 macrophages upregulate genes critical to extracellular matrix (ECM) remodeling while also synthesizing proteases, regulating fibroblast function [39,40], and modulating collagen deposition [41]. Cumulatively, these stabilizing processes converge to promote a sequence of pathological events, such as tumor cell proliferation, migration, and metastasis.

Interferon Regulatory Factors IRF4, IRF8, and IRF5 are transcription factors that regulate myeloid cell development (Figure 1), IRF5 is also important for M1 polarization [42].

### 2.3. Differential Reprogramming in Antitumor Therapy

M1–M2 macrophage polarization is a highly-regulated process contingent upon multiple signaling pathways, as well as transcriptional and posttranscriptional events [27,29]. Particular emphasis is reserved for inductive stimuli in the local microenvironment, which can be manipulated in vivo to favor a specific phenotype. When infection, injury, or inflammation increases in severity, polarization initially adopts the M1 conformation, leading to a systemic influx of TNF-α, IL1β, IL-12, and IL-23 to reconcile a homeostatic state. However, if this phase persists for a prolonged time, significant tissue damage can occur. Therefore, the prevalence of M1 macrophages is counterbalanced by a shift towards M2 expression, which induces the secretion of IL-10 and TGF-β to mitigate inflammation and drive reparative mechanisms [27,29]. Transient by nature, macrophage polarization is largely time- and tissue-dependent. Thus, it should be considered within this context rather than as a one-dimensional definitive analysis. As macrophage expression is inextricably linked to inflammation, resolving versus non-resolving inflammation must be distinguished. Resolving inflammation, which involves immune cell recruitment, tissue repair, and rapid renewal, can run its course in as little as a few days, all while leaving behind no consequential biological footprint. In chronic, non-resolving inflammation, as seen in select parasitical infections, can take up to several years to return to its natural state [43]. Nonetheless, the assumption of either macrophage phenotype and the time elapsed and morphology pathway/frequency favoring one as opposed to the other can reveal critical information about normal versus pathological events occurring at the intracellular level.

### 2.4. Tumor Microenvironment (TME)

The main driver in macrophage polarization events stems from the interactions amongst various cellular actors in the microenvironment [44]. While the TME consists of both cancerous and non-cancerous cells, most of these subpopulations cooperate synergistically to drive positive feedback loops conducive to tumorigenesis. An integrated network of lymphocytes, endothelial cell types, and fibroblasts are among the most prominent influences in establishing pro-carcinogenic conditions, which prime the subsequent development stages of tumor promotion, malignant conversion, and progression.

T lymphocytes (T cells), B lymphocytes (B cells), and natural killer (NK)/natural killer T (NKT) cells constitute the diverse lymphocyte presence in the TME. T cells, which include pro-tumor FOXP3+ regulatory cells, CD4+ Th2 helper cells, and Th17 cells, as well as antitumor CD8+ memory cells, CD4+ Th1 helper cells, and γδ T cells, account for up to 10% of the total tumor cell population, however, can also be found in substantial concentrations along the tumor periphery [44]. Although lesser in number than their T cell relatives, B cells typically found in lymphoid structures proximal to the TME exhibit this same functional duality, the morphology of which strongly correlates to cancer type. Lastly, innate NK and NKT cells inhabit the surrounding tumor region and drive cytotoxic mechanisms that compromise tumor survival.

Of the relevant endothelial cell types, lymphatic endothelial cells and vascular endothelial cells are prominent modulators of the TME. Lymphatic endothelial cells shape these conditions by either leveraging existing lymph conduits to drive tumor infiltration or actively inducing lymphangiogenesis due to macrophage- or malignant cell-produced VEGFC or VEGFD growth factors [45]. In addition, research delineates their role in influencing host tumor responses [46]. Similarly, vascular endothelial cells in the TME are recruited for tumor growth. However, angiogenesis, triggered by malignant cell signals or hypoxic conditions in the microenvironment, proceeds in an unregulated manner, resulting in irregular branching and uneven vasculature lumina. Moreover, the integrity of these vessels is substandard; their leaky properties disrupt interstitial pressure, blood flow, and nutrient delivery while worsening oxygenation in the TME and encouraging metastasis.

Lastly, any discussion of the major contributors to the TME would be incomplete without mentioning the role of myofibroblasts, also known colloquially as cancer-associated fibroblasts (CAFs). Typically aggregated along the cancer’s invasive boundaries, CAFs have been indicated in the synthesis of pro-tumor growth factors that trigger uncontrolled tumor cell division [47]. In addition, CAF-produced TGF-β causes the epithelial-mesenchymal transition, which amplifies immunosuppression and supports growth. The fibroblast-synthesized chemokine CXCL12 contributes to this phase by enhancing malignant cell prospects for survival and via the aggressive recruitment of additional progenitor cells into the TME GF-beta 1 attenuates the acquisition and expression of effector function by tumor antigen-specific human memory CD8 T cells [48]. Fibroblasts also assume a dominant role in establishing the TME by supplying structural ECM components and vital remodeling enzymes [49].

### 2.5. Tumor-Associated Macrophages (TAMs)

While the architects mentioned above of the TME command an important role in their contributions, TAMs merit particular emphasis. They possess a unique transcriptional profile distinct from M1 and M2 subtypes [50]; these stromal immunocytes directly infiltrate solid tumor tissues themselves or their immediate microenvironment. With the capacity to mimic both M1 and M2 roles, TAMs preferentially imitate M2 activity as a result of the suitable TME conditions in which they reside: cancer cells demonstrate elevated glycolysis, thereby acidifying the TME, activating regulatory macrophages, and subsequently increasing the supply of VEGF and arginase, all of which converge to induce the M2-aligned TAM characteristics [51]. Therefore, within the context of pathological disease states, TAMs typically function to inhibit pro-immunity signals while catalyzing each phase of cancer development within the TME.

**Figure 2:**
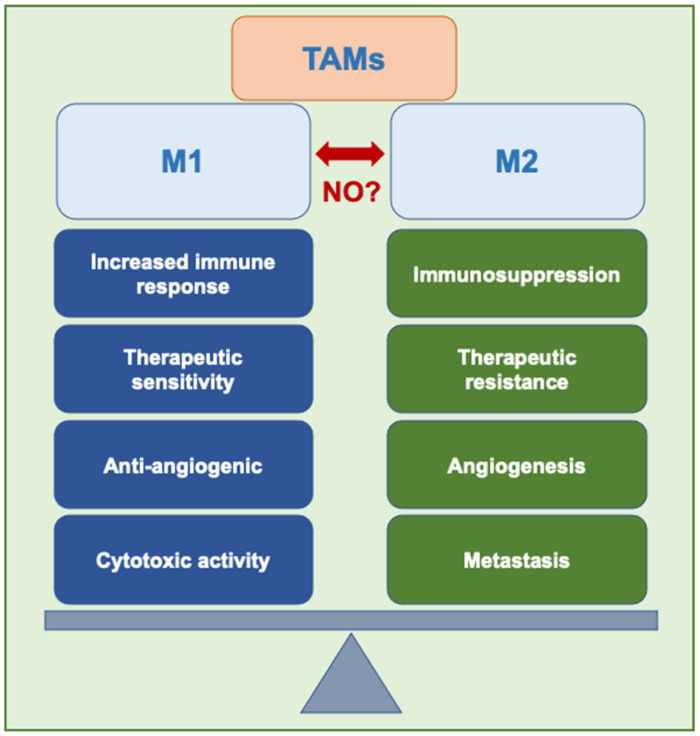
Repolarization of tumor-associated macrophages. M1-like and M2-like phenotypes shift expression depending on the microenvironment. Could iNOS-derived NO modulate TAMs?

Limited to the initiation phase, TAMs briefly favor M1-like distinction. At this stage, TAMs secrete signaling molecules containing growth factors, cytokines, chemokines, inflammatory substrates, and enzymes charged with effecting proteolysis. In addition, evidence suggests that these TAM-secreted growth factors function to nurture tumor stem cells (TSCs), the precursors to proliferative, self-renewing tumor cells. In reciprocation, TSCs induce monocyte differentiation towards the pro-tumor TAM phenotype [52]. Once tumors are introduced, the TAMs shift toward M2-type mechanisms that sustain sequential development [53] (Figure 2). In the progression stage, TAMs lead to the vascularization of solid tumor tissues. This network of blood vessels is critical for growth beyond a prescribed threshold—a phenomenon known as the “angiogenic switch”—in that it provides a means of ongoing oxygenation, nutrient delivery, and waste removal [54].

ECM modification describes the pathway preceding key metastatic events. TAMs are critical to this process as they synthesize TGF-β, which triggers EMT processes to improve motility and invasion. In addition, TAMs establish pre-metastatic niches even before tumor cell dispersion [55]. Features of this supportive environment include tumor-produced exosomes that direct and redirect myeloid cells to assume pro-tumoral roles through the upregulation of the MET receptor tyrosine kinase [56].

### 2.6. Macrophage Reprogramming

Given the high density of TAMs characteristic of most tumors, as well as its positive correlation to poor clinical prognoses, macrophages represent a promising target for immunotherapy intervention. As the chief modulator of the host immune response, in-vivo manipulation of their plasticity through programming and reprogramming of anti-inflammatory M2 to pro-inflammatory M1 phenotypes presents the opportunity to maximize tumoricidal defenses [57] and potentially improve clinical outcomes.

M1/M2 phenotypic expression shifts in response to inductive stimuli in the local microenvironment. When infection, injury, or inflammation increases in severity, polarization initially adopts the M1 conformation, leading to a systemic influx of TNF-α, IL1β, IL-12, and IL-23 to reconcile homeostasis. However, if this phase persists for a prolonged time, significant tissue damage can occur. Therefore, the prevalence of M1 macrophages is counterbalanced by a shift towards M2 expression, which induces the secretion of IL-10 and TGF-β to mitigate inflammation, enhance tissue repair, and promote vascularization [57,58].

STAT3 mediates the effects of anti-inflammatory cytokine IL-10. It is established that up-regulation of STAT3 is followed by down-regulation of pro-inflammatory cytokines IL-1, IL-12, TNF-α, and IFN-γ through IL-10-dependent signaling. STAT3 deficient mice show increased release of pro-inflammatory cytokines and IL-10 in response to stimulation by LPS. This indicates the potential of STAT3 in M1 to M2 reprogramming [59]. STAT3 was also found to lead to anti-inflammatory or pro-inflammatory responses depending on the signal. Studies on STAT3 induction in immunodeficient mice showed that anti-inflammatory M2 type polarization occurred when IL-10 was produced via transplanted regulatory T cells, whereas pro-inflammatory characteristics were obtained by activation with IL-6 and IFN-β [60].

IRF5 regulates M1 polarization and its expression is increased by GM-CSF, LPS, and IFN-γ [42]. IRF1 also facilitates M1 polarization. IRF1 and IRF5 cooperate towards M1-type polarization and also through IRF1′s ability to enhance IRF5 levels. However, IRF4 competes with IRF5 for MyD88 binding. IRF4 also antagonizes IRF5 binding to MyD88 and can promote M2 over M1 differentiation [61].

NF-κB activates the transcription of inflammatory genes. NF-κB transcriptional changes may shift cells from M1 to M2. In macrophages, p50 deficiency altered the activation of M2-specific promoters CCL17 and Arg-1 while up-regulating the transcriptional activation of M1-specific promoters, including iNOS, IFN-β, and TNF-α [62].

While recent studies have taken an optimistic outlook, it is important to be mindful of several challenges. First, TAMs have been implicated in inducing resistance to traditional antitumor interventions [58]. Additionally, rarely, TAMs act independently across cancers; for instance, in brain cancer, macrophages, and glioblastoma associate to elicit pathological states. Finally, cancerous cells have evolved significant advantages regarding evasion that make these targets challenging, such as the ability to thrive in hypoxic environments. This, taken together with their intrinsic self-renewing properties, present considerations that must be evaluated when designing appropriate therapies.

In terms of tissue-associated factors, differential microRNA (miRNA) exerts a profound influence on phenotypic expression. miRNAs control tumorigenesis by regulating inflammatory macrophage activation. miRNAs are non-coding single-stranded RNAs containing between 18 to 25 nucleotides, which are expressed in vertebrates and plants. They act as posttranscriptional gene expression regulators, fine-tuning various biological processes such as apoptosis, development, proliferation, and differentiation by binding to the 3′-untranslated region of the target mRNA and inhibiting its translation [63,64]. Current data strongly suggest that some miRNAs may be used as non-invasive biological markers for the early detection of cancer [65].

miRNAs play a key role in regulating TAM phenotypes in cancer [66]. Generation of miRNAs prompts M2-like TAM polarization and inhibits tumor infiltration of CD8^+^ cytotoxic T lymphocytes (CTLs) that enhances M1-like macrophage programming by producing IFN-γ, thus sustaining the immunosuppressive capacity of TAMs [66].

miR-222-3p, miR-145, miR-940, miR-103a, and let-7d promote the M2-like phenotype, whereas miR-155, miR-19a-3p, miR-18a, and let-7b promote the M1-like phenotype and have inhibitory effects on the M2-like phenotype, reviewed in [66]. For example, miR-155 expression is low in MRC1^+^ TAMs and inhibits tumor growth in a breast cancer mouse model by reprogramming M2-like macrophages toward classic M1-like activation [67].

Inducing the programming and reprogramming of anti-inflammatory M2 to pro-inflammatory M1 phenotypes presents the opportunity to maximize tumoricidal properties [57] and potentially compromise pathogenicity.

## 3. iNOS and Macrophages

The tumoricidal macrophages in the TME can actively produce NO [68,69]. The progression of the tumor is also affected by the anti- and protumor properties of NO of the tumor cells [70] Additionally, NO can modulate apoptosis and survival of various immune cells such as dendritic cells, mast cells, NK cells, macrophages, monocytes, Kupffer cells, microglia, eosinophils, and neutrophils [71]. With iNOS expressing antitumor macrophages, such modulation affects the immune response in particular with high NO levels [72]. The interplay of these effects may define a potent therapeutic target [73]. If the cell situation could be altered such that the TME’s low iNOS activity is reprogrammed to produce higher iNOS/NO concentrations, it may then be of therapeutic value [74,75].

Regulation of iNOS gene expression is known to be through the MAPK and NF-κB signaling pathways. In the NF-κB pathway, the p65-p50 dimer translocates to the nucleus from the cytoplasm to induce iNOS gene expression [76]. LPS binds to the TLR4 on the surface of macrophage membranes, leads to activation of MAPK or NF-κB signaling pathways and further iNOS gene expression [77]. In studies with RAW264.7 macrophages, LPS-mediated p65 NF-κB gene expression increased the iNOS expression and macrophages were polarized to the pro-inflammatory M1 state. Test compounds such as *Phyllolobium chinense* Fisch flavonoids could inhibit the iNOS transcription by inhibiting the activation of NF-κB signaling pathway, and consequently, macrophage M1 pro-inflammatory polarization, as observed in vitro [78]. Therefore, in a non-tumor environment, the downregulation of NF-κB/ iNOS/ NO is important in M1 polarization [78]. NF-κB signaling remains a key transcriptional regulator of both M1 and M2 polarization.

**Figure 3:**
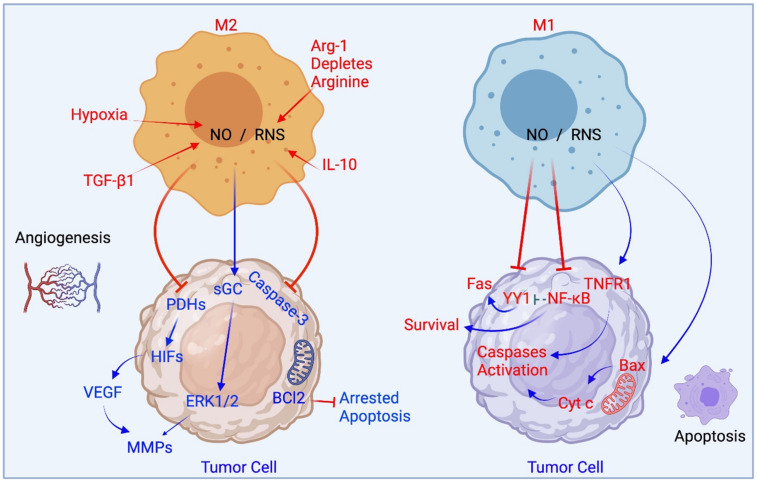
The role of M1-like and M2-like macrophages in apoptosis and angiogenesis.

In the tumor microenvironment, NF-κB pathways are involved in M1 polarization. Recent reports suggest oscillatory activation and sustained NF-κB signaling lead to distinct transcriptional responses in mouse macrophages [79]. M2 phenotype macrophages and their subsets produce low levels of NO/RNS. Microenvironmental conditions such as hypoxia and NO/RNS stabilize the transcription factor HIF and family members, activate the MAPK ERK1/2 pathways, and lead to the expression of VEGF and angiogenic promoting molecules. M1 phenotype macrophages produce high amounts of NO/RNS and promote apoptosis of tumor cells. This may be by modifying death receptor pathways, cytochrome c release from the mitochondria and by inhibiting NF-κB. NO from macrophage types M1 and M2 and their effects on apoptosis and angiogenesis are summarized in Figure 3. It is also established that the promoters of the iNOS gene include sites for AP-1. In M1 polarized macrophages, both AP-1 and NF-κB share several signaling pathways and transcription targets and can be activated by LPS, indicating cooperative activity [80].

The TNF receptor superfamily member 5 (TNFRSF5), also known as the co-stimulatory molecule CD40, is expressed on antigen-presenting cells, including subsets of monocytes, macrophages, and dendritic cells [63]. Antitumoral activity of TAM occurs by activation of the CD40L-CD40 pathway with agonists or with cells that express CD40L [64]. In pancreatic cancer animal models, treatment with anti-CD40 agonist led to the recruitment of macrophages into the tumor and their polarization toward ECM-degrading cells by inducing the release of IFNγ and CCL2 [81,82]. Antitumor activity of TAMs may be through NO. In iNOS-deficient macrophages such as in iNOS knockout mice, tumoristatic activity was of the anti-CD40 antibody-activated macrophages was demonstrated to be reduced though not completely abrogated [83]. This implies that NO is not the only mediator of the antitumor effects induced by αCD40-activated macrophages [83]. Another pathway promoting the antitumoral TAM activity involves induction of type I IFN or IFNα/β by TLR ligands, such as CpG DNA. Type I IFN has also been shown to enhance antitumor activities of myeloid cells [84].

## 4. Reprogramming between M1 and M2 States

### 4.1. The Proof Is in the TAM Pudding: Glioblastoma and iNOS

Glioblastomas are aggressive malignant brain tumors with poor prognosis and resistance to therapy [85]. The TME of glioblastomas consists of extracellular matrix, interstitial fluid, and non-neoplastic cells. The non-neoplastic cells in the TME of human GBMs are a large population of brain microglia and bone marrow-derived macrophages that constitute 30–50% of the total tumor cell mass, and both are collectively called TAMs [86]. As demonstrated in murine glioblastoma, the microglia rapidly change their morphology into an amoeboid-like shape, while circulating monocytes originating from the bone marrow invade the brain when a glioblastoma emerges and differentiate into macrophages and further become morphologically indistinguishable from microglia [87].

The TAMs in glioblastoma have great plasticity and diversity and express genes related to both M2 and M1 states that may break down L-arginine either via Arg-1 enzyme or iNOS, respectively, depending on the result on the pathway promoted [88,89,90]. M1 states show high expression of IL-12, IL-23, and a low expression of IL-10 and also produce high levels of pro-inflammatory cytokines IL-1β, TNF-α, and IL-6, and increase in the expression of iNOS and ROS [91]. Studies of TAMS in GBM examined with chlorogenic acid (or 5-caffeoylquinic acid), as a potential therapeutic approach, found a reduction in the growth of glioblastoma cells and in xenograft tumors by inhibiting the M2 polarization and promoting the M2- to M1- phenotype in xenograft models of mice and in co-culture studies of glioma cells and macrophages [92]. This occurred through the promotion of STAT1 activation and inhibition of STAT6 activation. More importantly, the level of IFNγ/LPS-mediated iNOS mRNA was upregulated while that of Arg-1 was down-regulated. Overall, apoptotic-like cancer cells were increased, and the growth of tumor cells was inhibited. In contrast, the proportion of CD11c-positive M1 macrophages increased in the tumor tissues, and the distribution of CD206-positive M2 macrophages was reduced [92].

Immunometabolism in glioblastoma is studied for the potential of modulation for the polarization of TAMs to M1 state or repolarization from M2 to M1. However, the balance within the human glioblastomas TME is mainly shifted to an overall tumor supporting state for these TAMs in light of the poor prognosis observed in glioblastoma associated with their accumulation [93]. Therefore, strategic mechanisms to induce iNOS-derived NO and ROS in TAMs may directly destroy tumor cells in glioblastoma, which could also include cytotoxicity through stimulating Th1 responses by the production of CXCL9 and CXCL10 and by generating cytotoxic CD8 cells via IL1β and TNFα [90,94]. As glioblastoma tumor cells are also very adaptive, arginine metabolism in the tumor cells has been examined and found to be very active in the tumor cells [95]. The presence of substantial arginine transporters in the pathway has allowed investigators to tamper with specific means to deplete arginine. Such was examined by generating a human recombinant PEG5000-Arg-1-cobalt derivative which caused arginine depletion in human glioblastoma cells and was found to be selectively cytotoxic to the cells [96] and later owed to long-term activation of autophagy of the tumor cells.

Inside the tumor mass, NO can be produced by both cancer cells and the TAMs. Therefore, the interplay of the cell situation and the environment and with possible induction or inhibition of iNOS/NO will determine the output of the anti- or pro-tumor effect. In glioma of GL261 murine- and U937 human studies performed in vitro with polarized macrophages, the M2 type TAM produced iNOS/NO and was found to protect the cancer cells from cisplatin-induced apoptosis and led to chemoresistance [97,98]. Here NO from the M2 state was found to activate cGMP/PKG pathway in the cancer cells and lead to phosphorylation and proteasomal degradation of syntaxin4. This led to inhibition of sphingomyelinase translocation to the plasma membrane and its activation, thus blocking cisplatin’s activity and providing cytoprotection by M2 derived iNOS/NO. As accepted by these and other investigators, M2-like macrophages show a reduced expression of iNOS and generate low NO levels that have cytoprotective action [97,98].

iNOS was found to have an important role in malignant glial transformation in vivo as an immunosuppressive mediator. Two iNOS inhibitors 1400 and S-MIU reduced tumor growth in a glioblastoma model of U87 cells overexpressing EGFRvIII variant [99]. As soluble factors produced by the tumor cells can also revert TAMs from M1 to an M2 phenotype despite pharmacological interventions, the interplay between glioblastoma cells and TAMs and reasons for the shift of metabolism is of interest within the TME. Finding ways to modulate the TME to antitumor roles may require examining more ways to gear TAMs towards the iNOS pathway and dissect crosstalk between tumor cells, TAMS, and T cells. Due to the plasticity nature of TAMs and their abundance within the glioblastoma TME, these remain as potential targets for repolarization and reprogramming to M1 antitumor states.

### 4.2. TAMs in Melanoma and iNOS

**Figure 4:**
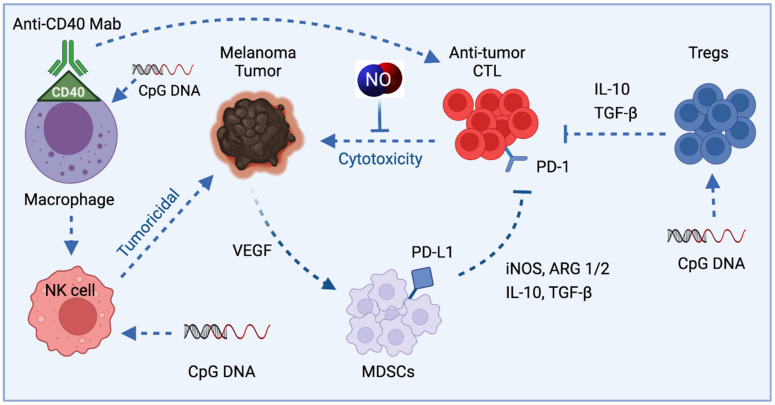
NO in melanoma tumor-induced immune suppression. Treatments such as CD40 activation and CpG may activate macrophages, produce NO and reduce immune suppression.

In melanoma, the tumors can directly suppress antitumor CTL by recruitment of suppressive immune cells regulatory T cells (Tregs) and myeloid-derived suppressor cells (MDSCs). MDSCs are a heterogeneous population of immature myeloid cells, which can strongly inhibit antitumor activities of T and NK cells and stimulate regulatory T cells (Treg), leading to tumor progression [100]. More recently, they have been found to be a major driver of an immunosuppressive tumor microenvironment [101]. MDSCs, in turn, can mediate suppression through the production of cytokines IL-10 and TGF-b, as well as through the production of Arg1 and iNOS. Direct suppression can occur through inhibitory ligands such as programmed death-ligand 1 (PD-L1), whose receptor, programmed death 1 (PD-1), is found on activated T cells (Figure 4).

NO or iNOS-derived NO is an immunosuppressive mediator, and in some cases, it leads to reduced tumor-inhibitory activity. In melanoma models, the autocrine effects of iNOS in γδT cells that secrete IL-17 lead to the recruitment of MDSCs [102]. In other melanoma-related studies, NO from MDSCs inhibit antigen presentation from dendritic cells to CD4+ T cells and demonstrates STAT1 nitration and inhibition of immune responses [103]. NO also blocks signaling through the IL-2 receptors of T lymphocytes by preventing phosphorylation of the intracellular signaling proteins STAT5, Akt, and Erk [104]. S-nitrosation of chemokines and nitration of tyrosine are mechanisms associated with the inhibition of T cells infiltration in tumors.

Tumor growth inhibition via macrophages occurs upon CD40 activation [105]. In melanoma models, macrophages activated in vivo by CD40 ligation using an anti-CD40 monoclonal antibody and co-cultured with B16 melanoma cells exhibited tumoristatic activity. High amounts of NO and TNF-α were produced [83,106]. The tumoristatic effect correlated with the level of NO production and was enhanced by LPS. Macrophage activation with anti-CD40 antibodies could be combined with other macrophage-activating agents, such as, CpG-containing oligodeoxynucleotides that may act by affecting the effector functions of TAMs to convert them from the M2 pro-tumor phenotype to M1 antitumor phenotype [106]. Combining anti-CD40 and CpG treatment in vivo similarly resulted in synergistic activation of macrophages and potent antitumor effects even in the absence of T cells, NK cells, or polymorphonuclear cells.

Short oligodeoxynucleotides that contain CpG motifs have immune-stimulatory properties [107]. They have successfully been used as adjuvants in several experimental systems [108]. CpG DNA influences macrophage activities. Macrophages exposed to CpG secrete the inflammatory cytokines TNF-α and IL-12 [109,110]. The IL-12 acts synergistically with CpG DNA to induce IFN-γ production by NK cells, causing further activation of macrophages [109,110]. Further, CpG DNA induces iNOS transcription upon pretreatment of macrophages with IFN-γ [110].

## 5. Therapeutic Approaches Utilizing Macrophage-Derived iNOS/NO in Cancer

Antitumor strategies targeting TAMs include potential mechanisms of lowering TAM survival, reducing macrophage recruitment, and switching M2-like TAMs into an M1-like phenotype [51]. Utilizing the iNOS derived-NO or exogenous NO delivery, there has been some success with nano-therapeutics. In the tumor microenvironment, the nanoparticles of self-assembled poly(L-arginine) are taken up by the activated macrophages, followed by the hydrolytic release of L-arginine, and conversion to NO by the iNOS of the TAM [111]. In low doses, the NO produced by this mechanism in tumor-bearing mice increased the angiogenesis of the tumor tissues, whereas the high doses led to tumor volume reduction and apoptotic tumor cell death [111].

Immunotherapeutic approaches have demonstrated reprogramming M2 to M1 macrophages [112,113]. As stated earlier, induction of the innate immune response is initiated by activating the macrophage to M1-type, which produces NO/RNS, secrete TNF-α, IL-1β, and IL-6 with pro-inflammatory cytokines proteases such as MMP-9. Higher NO production activates downstream signaling pathways that perform a critical role in the cytotoxic activity of immune cells against tumor cells [12,114]. Furthermore, among other immune cells, NO synthesis in NK cells was shown to regulate their tumoricidal activity to some extent [115].

### 5.1. iNOS Inhibitors

In colon, breast, gastric, hepatocellular carcinoma, melanoma, ovarian, leukemia, gastric, prostate, esophageal, and cervical cancers, high iNOS expression has correlated relatively well with poor patient survival [116,117]. Thus, iNOS expression may be used as a biomarker of poor patient prognosis and perhaps survival [118,119]. In contrast, a favorable prognosis has been associated with high iNOS expression in ovarian [120] and non-small cell lung cancers [121].

When cell lines expressing high levels of iNOS, such as that of triple-negative breast cancers, were treated with 1400W, which is a highly selective iNOS inhibitor, or L-NAME, which is a relatively selective eNOS inhibitor, or L-NMMA, which is pan-NOS inhibitor, they all reduced cell proliferation, migration, and mammosphere formation [122]. In a xenograft model of TNBC, treatment of mice with L-NAME, and L-NMMA significantly reduced tumor growth [122]. Administration of AG, another iNOS specific inhibitor to athymic nude mice bearing TNBC xenografts, abated tumor growth and metastatic burden [123]. Further, the growth of glioma [124] or melanoma [125] cells in xenografts was significantly reduced when iNOS was silenced in these cells before they were implanted. The overarching data from all of these studies is the observation that the enhanced growth of the iNOS-overexpressing tumors appears to be due to enhanced angiogenesis, reviewed in [3].

iNOS deficient mice exhibited enhanced M1 macrophage polarization with no significant effects on M2 macrophages. L-NIL, an iNOS selective inhibitor, significantly enhanced M1 macrophage polarization in cell cultures from wild-type (WT) mice. Whereas the NO donor SNAP, suppressed M1 macrophage differentiation in WT and *iNOS^−/−^* cell cultures [126].

### 5.2. NO and Curcumin: A Natural Dietary Compound

Natural dietary compounds modified or formulated as nanoparticles induce iNOS in TAMs and show promise in reprogramming M2 to M1 states to produce antitumor effects [127]. A curcumin formulation containing two additional natural polyphenols, aptly named TriCurin, produced repolarization of M2 TAM that had higher Arg-1 expression into the M1 TAM population with higher iNOS expression. The underlying mechanism appears to be the suppression of activated STAT3 in M2 type TAM, which causes activation of STAT1, leading to the M1 phenotype. Co-activated transcription factors STAT1 and NF-κB initiate the expression of the iNOS and NO in M1 cells leading to tumor elimination [128,129]. Of note, these M1 phenotypes are low-IL10 and high-IL-12 and showed anti-glioblastoma activity [130]. Specifically, for TriCurin, the M1 TAM-derived IL-12 that was induced was responsible for the recruitment of NK cells and cytotoxic T lymphocytes, leading to the reduction in cervical cancer cells in xenograft tumors [129].

### 5.3. NO and Immunomodulation with microRNAs

**Figure 5:**
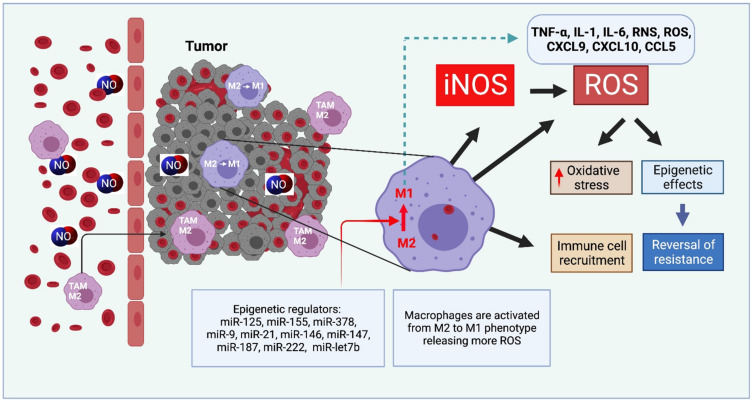
Exogenous NO or iNOS-derived NO modulate the macrophage status. M2-type macrophages may be re-polarized into M1 phenotype via regulatory miRNA. ROS-generated oxidative stress may produce a cytocidal profile and reverse tumor progression.

MicroRNA-mediated regulation modulates macrophage states to the M2 or M1 phenotype [131]. Delivery with nanomaterials has been developed wherein a marked increase in iNOS expression occurs for inducing or reprogramming the TAMs to M1-state or repolarization from M2- to M1-state, which provides an antitumor immune response (Table 1). For example, Zhang et al. [128] designed lipid-coated calcium phosphonate nanoparticles which were further conjugated with mannose for specific delivery of miR155 to TAMs, which altered phenotype successfully from pro-tumor M2-like TAMs to antitumor M1-like TAMs, and therefore produced a potent antitumor immune response and inhibiting tumor growth, reviewed in [132]. Layered double hydroxides NPs that are miR-155 loaded are taken up by TAMs, increase their iNOS expression, decrease the expression level of phosphorylated STAT3 and ERK1/2 and activate NF-κB expression. Combined therapies showed improvement also, for example, these nanoparticles with carboplatin improved TC-1 tumor recession in animal models and prolonged overall survival [133]. Similarly, Parayath et al. [134] developed a CD44 targeting hyaluronic acid-poly(ethylenimine) (HA-PEI)-based nanoparticle for the delivery of miR-125b to peritoneal macrophages to promote M1-like TAMs activation in the lungs. In vivo results found a more than 6-fold increase in the ratio of M1 to M2 TAMs and a 300-fold increase in iNOS to Arg-1 in TAMs after treatment with HA-PEI-125b nanoparticles. The continued success of inducing M1-like TAMs polarization opens many avenues in anticancer immunotherapy via NO and enhancement of ROS release (Figure 5).

### 5.4. NO-Releasing Nanoparticles

Synthetic compounds and various carriers also hold promise considering that years of iNOS focus have created avenues to mimic iNOS/ NO activity in various ways [1]. Nanoparticles are demonstrated to preferentially accumulate in macrophages after systemic administration [135]. Nanoparticles made to release NO produce cytotoxicity depending on the nanocarrier’s chemical nature, the concentration of NO released and the cell type. Low concentrations of NO released will have a proliferative effect on tumor cells, whereas high NO flux is expected to have toxic effects. There is considerable interest in identifying delivery methods to modulate TAM polarization for cancer treatment. TAMs overexpress the macrophage mannose receptor and therefore, mannose functionalized nanoparticles are used for recognition and internalization [136]. Affinity to TAMs was improved via a mannose- conjugate modified on lipid-coated calcium phosphonate nanoparticles which delivered miRNA into TAMs in vitro [137]. Some NO-releasing nanoparticles and materials with potential use in cancer treatment are presented in Table 1 and principal actions are discussed. Reviews of various types of nanoparticles and effects on TME are found in, [135,138,139].

Certain NO-releasing nanoparticles have been designed for photo-release. Nanoparticles of supramolecular assemblies of cyclodextrin-based polymer contain a NO photo donor and a fluorophore/photochrome dyad with an average size of 30 nm releases NO by light input [140]. Photogenerated NO in human melanoma cancer cells showed cytotoxicity with light stimulation and low levels of cytotoxicity in the dark. Similarly, internalized nanoparticles NONOate into the endosomes and lysosomes of a cell, and cytotoxicity from NO-mediated apoptosis was found with NONOate modified silica nanoparticles which produced high NO flux [141]. However, the desired sustained NO release akin to endogenous production by iNOS/ NO was not obtained.

Gold nanoparticles with 2-mercapto-5-nitrobenzimidazole also photo release NO and a low dose has similar antitumor and cytotoxicity effects as cisplatin in HeLa, Siha (cervical cancer cell lines), MCF-7 (breast cancer cell lines), and A549 (lung cancer lines); wherein 80% lower dose of Gold nanoparticles was found to produce cytotoxicity as that of 10 g/mL of cisplatin [142].

**Table 1:** Nanoparticles releasing NO or inducing iNOS and effect on macrophages or TAM.

Nanoparticles and Effect on iNOS or NO	Model System or Cell Type	Effect	Reference
CD44 coated HA-PEI based NPs, miR-125b loaded, iNOS increased	Naïve and KRAS/p53 double mutant nonsmall cell lung cancer (NSCLC) mouse model	Specifically target peritoneal macrophages which reprogram lung TAMs into M1 type	[143]
Layered double hydroxides NPs, miR-155 loaded, acidity sensitive, taken up by TAM iNOS increased	TC-1 mouse tumor model Uptake by TAM Repolarize TAM into M1	Synergistic enhancement of therapeutic effects with programmed cell death-1 antibody (α-PD-1) antibody	[133]
Lipid-coated calcium phosphonate, miR-155 conjugated mannose, iNOS increased	S180 mouse sarcoma model	Repolarize M2 into M1 TAMs Significant antitumor effect	[137]
Gold nanoparticles, Photo release of NO	HeLa	Low doses of Gold nanoparticles were found to produce cytotoxicity as that of 10 g/mL of cisplatin	[142]
Poly(D,L-lactic-co-glycolic) acid (PLGA), loaded with ruthenium nitrosyl compounds, NO releasing upon light irradiation	Melanoma B16-F10 cells	In vitro cytotoxicity assays showed cell death	[144]
Cyclodextrin and NO photorelease by a donor	HeLa, Melanoma, A431- Human squamous carcinoma, Melanoma	Phototoxicity cell mortality	[145] [146] [147]
Polymeric, NO-releasing	BE(2)-C, Neuroblastoma cell line	Cisplatin in combination with nanoparticles produced synergistic cytotoxicity	[148]
4-arm branched polymer, NO-releasing	Human head and neck cancer cell line human breast cancer cell lines	Improved cell mortality	[149]
Liposome, NO-releasing	Breast cancer cell lines MDA-MB-231 and MDAMB-468	Improved cell mortality	[150]

In addition to NONOates, S-nitrosothiols (RSNOs) such as S-nitrosoglutathione (GSNO) were encapsulated into polymeric nanoparticles. GSNO was incorporated into polymeric nanoparticles consisting of diblock copolymers, which extended the RSNO stability. Because the combination of NO donors with classical chemotherapy agents is of considerable interest, GSNO-containing polymeric nanoparticles and cisplatin were used in in vitro experiments [148]. NO-polymeric nanoparticles showed enhanced NO stability in aqueous media, were non-toxic and could efficiently release NO intracellularly [148]. Neuroblastoma cell lines treated with GSNO-containing polymeric nanoparticles followed by cisplatin provided sensitization of cells and lower IC50 of cisplatin. NONOate-multiarm polymer nanocarriers to tumor-bearing nude mice inhibited tumor growth and extended the average survival of the animals in 7 weeks compared with intravenous administration of the classical NO-donor prodrug JS-K, and owed to a steady NO release profile. In vivo models may be reexamined for effects on macrophages [148].

It is important to note that nanoparticle-based studies on the release of NO and the effects on the TME or TAMs should use appropriate reference treatment conditions for accurate comparisons of the efficiency of nanoparticle-induced effects. Nanoparticle-induced macrophage programming effects are generally compared to small molecule-induced effects. Some anticancer drugs may suppress immune activity within tumors and promote tumor growth and continue to be used in the clinical landscape. Therefore, it is imperative to compare the effect of nanoparticle-treated macrophages to biomolecule-treated macrophages, for example, with biomolecules such as IFN-γ, IL-4, IL-10, or LPS, or drug-treated macrophages for advancing the field immunotherapy based on NO.

## 6. Perspectives and Conclusions

From what we understand so far about: (i) pro- and antitumor properties of NO, (ii) the tumorigenic and consistent NO production by the tumor cells [1,12], (iii) NO donors and nanoparticles producing high levels of NO that has antitumor activity, and (iv) the tumoricidal M1 producing NO, is that combining these effects and modulating iNOS may produce a stronger therapeutic effect [75,98,151]. NO-mediated enhancement of immunotherapies is possible as some biologics are found to increase NO levels. For example, antibodies that target the overexpressed programmed cell death-1 signaling cascade member PDL-1L ligand (atezolizumab, avelumab, and durvalumab) of cancer cells, or antibodies that block the receptor PD-1 on the immune cells such as natural killer T cells, dendritic cells, and B cells, CD4+ and CD8+ cells [152] can benefit from NO modulation as a combined therapy. As a result, these therapies indirectly increased NO release in patients. Furthermore, as the transcription factor YY1 further enhances PD-1L expression, and NO is known to inhibit YY1; therefore, an NO donor can further improve the effects of PD-1/PDL-1L immunotherapy [153].

Macrophage polarization of M2 states exists as a continuous spectrum [154]. Reprogramming of macrophages can change their functional phenotype as the microenvironment changes [155]. Another state besides M1 and M2 is also proposed, currently referred to as the switch M3 state which has more aggressive M1 activity [156]. It may be envisioned that partial depolarization or an incomplete polarization may be a strategy to produce other reprogrammed states of activated TAM functions with specific functional phenotypes supporting or against tumor growth. Switching phenomena have been proposed for other disease types and may include cancer as well [156]. Understanding the mechanisms of macrophage reprogramming and iNOS-mediated processes in TAMs and tumor cells will pave the way in identifying and selecting new therapeutic targets against cancer.

## Abbreviations

ECM	extracellular matrix
GM-CSF	granulocyte–macrophage colony-stimulating factor
IFN-γ	interferon-γ
IFN-γ	interferon-γ
IL-4	interleukin-4
IL-8	interleukin-8
JAK/STAT	Janus Kinase
LPS	lipopolysaccharide
MAPK	mitogen-activated protein kinase
miRNA	microRNA
MMPs	matrix metalloproteinases
Mrc1	mannose receptor C1
NO	nitric oxide
NOS2 or iNOS	nitric oxide synthase 2 or inducible NOS
PBMCs	peripheral blood mononuclear cells
PDGF	platelet-derived growth factor
PI3K	Protein Kinase B-phosphatidyl-inositol-3-kinase
ROS	reactive oxygen species
STAT1	signal transducer and activator of transcription 1
TAMs	tumor-associated macrophages
TGF-β	transforming growth factor-β
Th1 cells	type I helper T cells
TLR	toll-like receptor
TLR4	toll-like receptor 4
TME	tumor microenvironment
TNF-α	tumor necrosis factor-α
VEGF	vascular endothelial growth factor
